# Generative and Foundation-Based Artificial Intelligence in Medical Imaging: A Bibliometric Analysis of Global and United Kingdom Research, 2017-2025

**DOI:** 10.7759/cureus.113726

**Published:** 2026-07-31

**Authors:** Jatin Naidu, Sonia Naidu, Vasanth Baskaradoss

**Affiliations:** 1 Radiology, Lewisham and Greenwich NHS Trust, London, GBR; 2 Surgery and Interventional Science, University College London, London, GBR; 3 Obstetrics and Gynaecology, University College London Medical School, London, GBR; 4 Radiology, Kettering General Hospital NHS Foundation Trust, Kettering, GBR

**Keywords:** artificial intelligence, bibliometrics, generative artificial intelligence, medical imaging, united kingdom

## Abstract

Artificial intelligence (AI), including generative and foundation-based methods, has rapidly expanded within medical imaging research, but the structure, citation impact, collaboration patterns, and thematic orientation of the United Kingdom national research ecosystem remain incompletely characterised. This bibliometric analysis examined Scopus-indexed publications from 2017 to 2025 using a predefined search strategy targeting generative and foundation-based AI methods in medical imaging. Records were analysed globally and filtered for United Kingdom affiliation. Descriptive indicators, including total publications, total citations, citations-per-paper, and year-on-year growth, were calculated. Co-authorship and keyword co-occurrence networks were generated using VOSviewer version 1.6.19. A total of 13,452 publications were identified globally, receiving 194,650 citations and a global citations-per-paper value of 14.47. Of these, 889 publications were United Kingdom-affiliated, representing 6.6% of global output. The United Kingdom ranked fourth by publication volume yet demonstrated higher unadjusted citations per paper than several higher-volume countries, with a value of 21.00. United Kingdom output increased approximately 18-fold between 2017 and 2025, with evidence of a citation-lag effect in recent years. The leading UK institutions had the highest full-count publication totals, although institution-level counts were not mutually exclusive because individual publications could be attributed to multiple organisations. Countries with more journal-dominant dissemination profiles also had higher unadjusted citations-per-paper values, although this descriptive comparison could not determine whether document-type composition explained the differences. Keyword analysis identified three principal thematic clusters: generative and deep learning methodologies, MRI- and diffusion-focused applications, and broader diagnostic imaging workflows. More recent highly cited publications included diffusion- and foundation-model architectures, although citation-age differences limit temporal interpretation. United Kingdom-affiliated research represents a rapidly expanding and highly cited component of the global generative and foundation-based AI in medical imaging literature. These findings provide a transparent bibliometric reference point for monitoring research activity, collaboration patterns, and potential translational priorities while recognising that citation-based indicators do not directly measure clinical implementation, methodological quality, or real-world impact.

## Introduction and background

Artificial intelligence (AI) refers broadly to computational systems capable of performing tasks that have traditionally depended on human expertise, including pattern recognition, prediction, and decision support [1]. Advances in machine learning and deep learning have substantially expanded the capacity of AI systems to learn complex representations from large datasets, driving rapid research activity across many areas of healthcare. Within medical imaging, AI research has explored applications in image acquisition, reconstruction, segmentation, synthesis, triage, and interpretation, with the potential to support diagnostic efficiency and consistency [2]. These developments have positioned AI as a central area of investigation in radiology and other imaging-intensive specialities.

In recent years, generative AI has emerged as a particularly influential class of methods within medical imaging. Generative models, including generative adversarial networks (GANs), diffusion models, and variational autoencoders, are designed primarily to generate or transform data, such as synthesising images, reducing noise, or translating between imaging modalities. Foundation models, by contrast, are large-scale models pretrained on broad datasets and subsequently adapted to multiple downstream tasks; they may be generative, discriminative, or multimodal. Generative and foundation-based architectures have therefore enabled image synthesis and transformation while also supporting more generalisable applications across imaging tasks [3]. These methods have been applied to accelerated magnetic resonance imaging reconstruction, image enhancement, data augmentation, anomaly detection, multimodal image synthesis, image interpretation, and report generation [4,5]. Collectively, they extend beyond conventional task-specific discriminative modelling and support broader manipulation, representation, and interpretation of imaging data.

The rapid growth of generative and foundation-based AI research has produced a large, heterogeneous body of literature spanning clinical journals, engineering venues, and conference proceedings [6]. As this field expands, it becomes increasingly important to understand how research activity is distributed, which countries and institutions contribute most substantially, how collaborative networks are structured, and which thematic areas dominate scholarly output. Such insights are essential for informing research strategy, funding allocation, and translational planning [7].

Bibliometric analysis provides a systematic and reproducible framework for quantitatively assessing research output, citation impact, collaboration patterns, and thematic evolution within large scientific corpora. By applying mathematical and statistical techniques to bibliographic metadata, bibliometric methods enable the identification of influential publications, emerging research topics, and structural relationships among authors, institutions, and countries [8]. Previous bibliometric studies have examined AI broadly across medicine and radiology. Chogtu et al. evaluated the bibliometric development of AI in radiology during 2018-2022 [9]. Lin et al. analysed the wider medical AI literature from 2019 to 2023, encompassing areas such as digital health, precision medicine, public health, and medical imaging [10], while Baalla and Jellouli mapped the broader medical imaging AI literature, with particular emphasis on hospital performance, governance, and regulation [11]. However, these analyses treated AI as a broad technological field and did not specifically delineate the rapidly emerging literature on generative AI and foundation-based methods, including GANs, diffusion models, large language models, vision-language models, and general-purpose foundation models. They also did not systematically compare UK research performance with the global literature as we do in the present study.

The United Kingdom represents a particularly relevant context for such an analysis. The UK hosts a strong academic medical imaging community, maintains a nationally integrated healthcare system, and has articulated strategic priorities for responsible AI development and deployment in healthcare [12,13]. These features position the UK as both a major contributor to methodological innovation and a relevant context for translational research and evaluation.

A global bibliometric analysis by Chogtu et al. identified England among the leading contributors to AI research in radiology but assessed its publication output only as part of an international country-level comparison and did not examine UK institutions, collaboration networks, citation performance, or thematic structure in detail [9]. More recently, a bibliometric study evaluated generative AI research in healthcare imaging in India [14]. However, to our knowledge, no study has provided a detailed characterisation of the UK research ecosystem or directly examined its position within the global literature on generative and foundation-based AI in medical imaging. Therefore, this study undertook a comprehensive global bibliometric analysis alongside a focused assessment of UK-affiliated research, including publication output, citation impact, institutional productivity, collaboration patterns, and thematic development.

Accordingly, the present study aims to conduct a bibliometric analysis of United Kingdom-affiliated research in generative and foundation-based AI in medical imaging between 2017 and 2025. The year 2017 was selected to capture the early expansion of modern generative approaches in medical imaging, particularly GAN-based methods, while allowing the subsequent emergence of diffusion models, large language models, and foundation-model architectures to be examined within the same longitudinal period. The endpoint of 2025 represented the most recent complete calendar year available before the Scopus dataset was exported on 6 February 2026. By examining publication trends, citation impact, country and institutional contributions, collaboration networks, and keyword co-occurrence patterns, this study seeks to map the structure and evolution of this research domain and to contextualise the UK contribution within the global literature. These findings provide an empirical baseline to support future research planning and policy discussion while recognising that bibliometric indicators do not directly establish clinical translation or implementation. This article was previously posted to the medRxiv preprint server on June 29, 2026.

## Review

Materials and methods

Search Strategy

The Scopus database was used to identify relevant publications because of its broad coverage of biomedical, engineering, computer science, and conference proceedings literature, which are important dissemination routes for AI research in medical imaging. Searches were conducted in the title, abstract, and keyword fields for publications indexed between 2017 and 2025. No restrictions were applied by language or document type.

The search combined terms related to generative and foundation-based AI with those related to medical imaging. It was designed to capture the principal methodological categories represented during the study period, including GANs, diffusion models, foundation models, large language models, ChatGPT, and Gemini AI. The complete Boolean search strategy for our global search was:

TITLE-ABS-KEY("generative AI" OR "generative artificial intelligence" OR "generative adversarial network*" OR GAN OR "diffusion model*" OR "foundation model*" OR "large language model*" OR ChatGPT OR "Gemini AI") AND TITLE-ABS-KEY("medical imag*" OR "diagnostic imaging" OR radiolog* OR CT OR MRI OR ultrasound OR "computed tomography" OR "magnetic resonance") 

The strategy used broad umbrella concepts and the dominant model families represented across the study period rather than an exhaustive list of architecture-specific terms. The terms “generative AI”, “foundation model*”, and “large language model*” were intended to capture the wider field, while GANs and diffusion models were included separately because of their established prominence in medical imaging. The UK dataset was generated by applying LIMIT-TO (AFFILCOUNTRY, "United Kingdom") as stated below:

TITLE-ABS-KEY("generative AI" OR "generative artificial intelligence" OR "generative adversarial network*" OR GAN OR "diffusion model*" OR "foundation model*" OR "large language model*" OR ChatGPT OR "Gemini AI") AND TITLE-ABS-KEY("medical imag*" OR "diagnostic imaging" OR radiolog* OR CT OR MRI OR ultrasound OR "computed tomography" OR "magnetic resonance") AND (LIMIT-TO (AFFILCOUNTRY, "United Kingdom"))

The search was executed, and the datasets were exported on 6 February 2026. As Scopus is continuously updated, subsequent execution of the same query may produce slightly different record counts. All analyses were based on the datasets exported on 6 February 2026.

Eligibility and Screening

Publications were eligible if they were indexed in Scopus between 2017 and 2025 and retrieved by the predefined search strategy. This required at least one AI term and at least one medical imaging term to appear in the Scopus title, abstract, or keyword fields. All Scopus-indexed publication languages and document types were eligible.

For inclusion in the UK dataset, at least one author affiliation had to be indexed by Scopus as being located in the United Kingdom. First author or corresponding author status was not required. Non-English publications were retained where they met the search and affiliation criteria and contained sufficient indexed metadata for the relevant analysis.

Eligibility was determined by the predefined Scopus query and, for the UK analysis, the affiliation country filter. No manual title or abstract screening or additional relevance exclusions were undertaken after retrieval.

To assess the precision of the search strategy, a stratified random sample of 400 records was selected from the complete search results. Records were stratified by publication year into pre-2023, 2023, 2024, and 2025 groups, with proportional allocation reflecting the distribution of records across these periods.

Each sampled record was manually reviewed and assigned to one of four mutually exclusive categories according to its principal methodological focus: generative AI in medical imaging, foundation-based AI in medical imaging, potentially relevant, or unrelated. Records were classified as potentially relevant when the title, abstract, or indexed keywords indicated a plausible application of generative or foundation-based AI to medical imaging, but the available bibliographic information was insufficient to confirm that this represented the principal methodological focus of the publication.

Search precision was estimated using two definitions. The primary strict definition treated only records classified as generative AI or foundation-based AI in medical imaging as relevant. A secondary inclusive definition additionally treated potentially relevant records as relevant. Wilson 95% confidence intervals were calculated for each classification category and for both precision estimates. The validation exercise was used to assess the performance of the search strategy and did not result in record-level exclusions from the bibliometric datasets.

Data Cleaning and Normalisation

Bibliographic metadata were exported from Scopus, including publication year, document type, author names and identifiers, affiliations, citation counts, funding information, and indexed keywords. Exported records were checked for the completeness of variables required for each planned analysis.

Because Scopus was the sole bibliographic database, cross-database duplicate removal was not required. Potential duplicate records within the exported datasets were checked using Scopus record identifiers, DOIs, titles, and publication years. Records with incomplete metadata were excluded only from analyses requiring the missing variable and were retained for all other applicable analyses.

Funding-body names and counts were retained as indexed by Scopus and were not hierarchically consolidated. Consequently, UK Research and Innovation and its constituent councils, including the Engineering and Physical Sciences Research Council and Medical Research Council, could appear as separate entities, and their counts were not combined.

Institutional affiliations were generally retained as indexed by Scopus. However, directly identifiable hierarchical duplicates within the reported productivity ranking, such as the University of Oxford and University of Oxford Medical Sciences Division, were consolidated under the parent institution to avoid presenting the same institutional structure as separate ranked entities. No comprehensive institution-wide normalisation was otherwise undertaken. Organisations with incomplete or unresolvable affiliation metadata were not assigned to a separate institutional category.

Generic indexing terms, including “article”, “human”, and “humans”, were removed before keyword-network analysis. No formal VOSviewer thesaurus file was applied. Synonymous terms, such as “MRI” and “magnetic resonance imaging,” were therefore retained as separate terms when indexed separately by Scopus.

Author identities were determined using Scopus author identifiers and indexed author names. No additional manual verification or disambiguation using ORCID records, institutional profiles, or personal webpages was undertaken.

Bibliometric Indicators

Descriptive bibliometric analyses were performed using Microsoft Excel (Microsoft Corporation, Redmond, WA, USA). The principal indicators were total publications, total citations, citations per paper, and annual publication growth.

Citations per paper were calculated as total citations divided by total publications. Year-on-year growth in UK publications was calculated as:

\[ \left[\frac{\mathrm{UK}\ TP_{n}-\mathrm{UK}\ TP_{n-1}}{\mathrm{UK}\ TP_{n-1}}\right]\times 100 \]

where UK TPₙ represents the number of UK-affiliated publications in the current year and UK TPₙ₋₁ represents the number in the preceding year.

Historical annual citation counts for highly cited publications were obtained using the Scopus analyze search results function by sequentially updating the date filters for each relevant year. Citation counts used in the highly cited publication analysis represented citations accrued through 31 December 2025. The approximate annual citation rate was calculated by dividing total citations by the number of calendar years from the publication year through 2025 inclusive (2025−publication year+1). This year-based calculation did not account for the exact publication month or date and was therefore interpreted as an approximate rather than exact annualised rate. For publications with a complete five-year citation window, mean annual citations during the first five calendar years were calculated by summing citations accrued in the publication year and the subsequent four calendar years and dividing by five.

Full counting was used for country- and institution-level publication indicators. Each publication was credited to every contributing country and every Scopus-indexed institutional affiliation represented in its author affiliations. Multinational publications were therefore attributed to each unique participating country. As a result, country and institutional publication counts were non-mutually exclusive and could exceed the number of unique publications when summed. Country percentages could similarly sum to more than 100%.

VOSviewer Network Analysis

Keyword co-occurrence and author co-authorship analyses were performed using VOSviewer version 1.6.19, released on 31 October 2023 and developed by Nees Jan van Eck and Ludo Waltman at Leiden University’s Centre for Science and Technology Studies.

Networks were constructed using association strength normalisation and full counting. Full counting was selected because the analysis aimed to describe participation and network visibility, with each publication credited fully to every contributing author or keyword. For both networks, a fixed seed of 1 was used for the advanced layout and clustering parameters; complete layout and clustering settings are provided in Supplementary Material 1.

For author co-authorship mapping, documents with more than 25 authors were excluded. Authors were required to have contributed to at least three documents. Among authors meeting these criteria, the 50 authors with the highest total link strength were retained for network construction.

For keyword co-occurrence analysis, a minimum occurrence threshold of 10 was applied. Where more than 40 keywords met this threshold, the 40 keywords with the highest number of occurrences were retained for network construction.

VOSviewer clusters were generated algorithmically according to the strength of co-authorship or keyword co-occurrence relationships. Cluster themes were subsequently assigned qualitatively by reviewing the titles, abstracts, and keywords of publications associated with the most prominent authors or terms within each cluster. The resulting labels were interpretive summaries produced by the study authors and were not generated automatically by VOSviewer.

The Scopus dataset and VOSviewer files supporting the analyses are available in the accompanying Zenodo repository: https://doi.org/10.5281/zenodo.18659245.

Results

Dataset Identification and Search Strategy Validation

**Figure 1:**
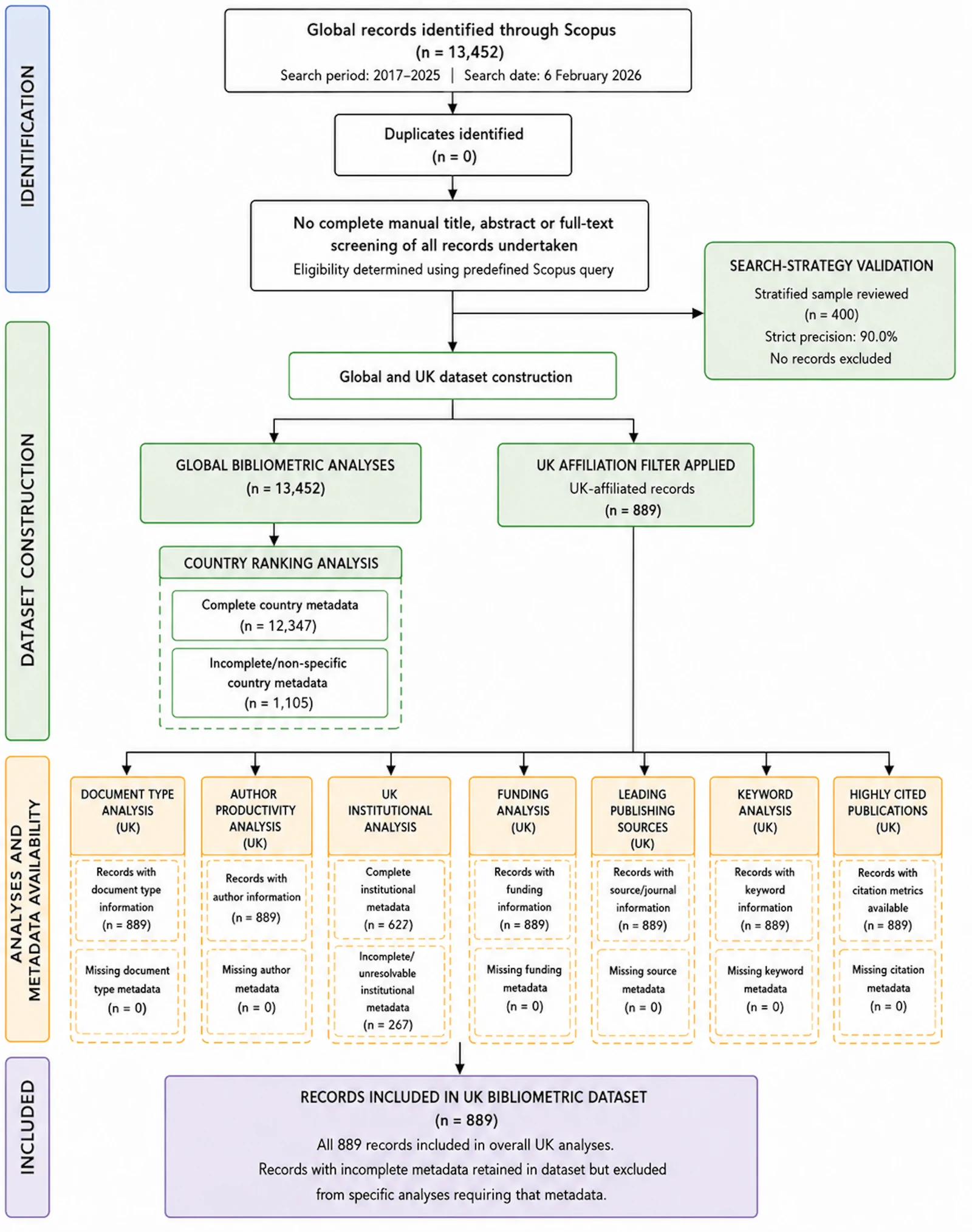
PRISMA style flow diagram of bibliometric record identification, search strategy validation, dataset construction, and analysis-specific metadata availability Blue indicates record identification; green indicates dataset construction and search strategy validation; orange indicates analysis-specific metadata availability; and purple indicates the final UK bibliometric dataset. Dashed borders denote metadata completeness assessments within individual analyses. The figure was created using Canva (Canva Pty Ltd, Sydney, New South Wales, Australia). n, number of records; PRISMA, Preferred Reporting Items for Systematic Reviews and Meta-analyses; UK, United Kingdom.

The predefined Scopus search identified 13,452 records published between 2017 and 2025, of which 889 had at least one United Kingdom affiliation (Figure 1). No duplicate records were identified.

Among the 400 records included in the stratified validation sample, 360 were classified as definitively relevant, giving a strict precision of 90.0% (95% CI: 86.7%-92.6%). A further 36 records were classified as potentially relevant and four as unrelated. When potentially relevant records were included, the estimated relevance proportion was 99.0% (95% CI: 97.5%-99.6%). The validation exercise was not used to exclude records from either dataset.

Overall Publication Trends

**Figure 2:**
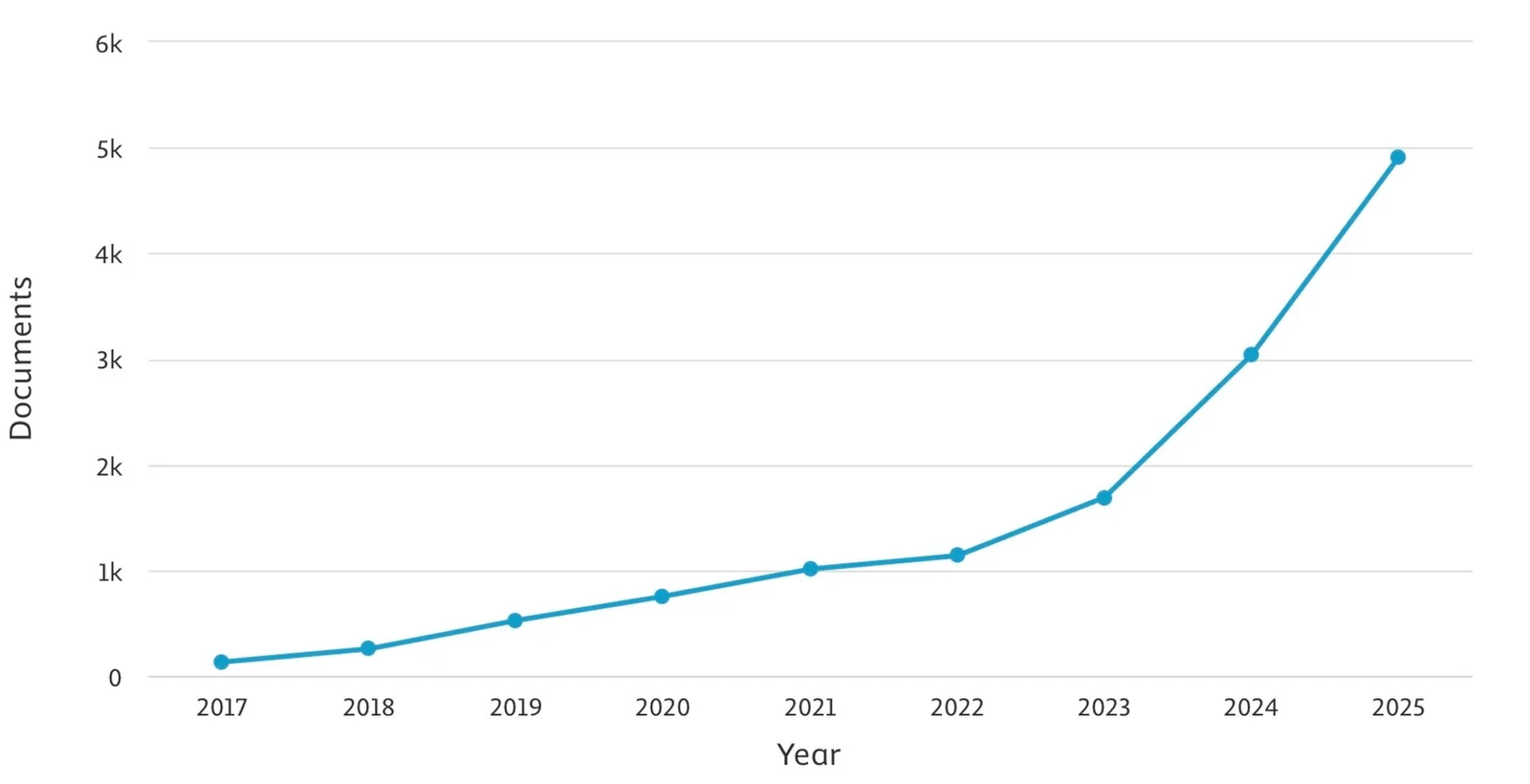
Annual global publication trends in generative and foundation-based artificial intelligence applied to medical imaging (2017-2025) The line plot shows the number of Scopus-indexed publications per year. The chart was generated using Microsoft Excel for Microsoft 365, 2026 (Microsoft Corporation, Redmond, WA, USA), from bibliographic data exported from Scopus.

Global publication output increased substantially across the study period, particularly after 2022 (Figure 2). The complete global dataset comprised 13,452 publications and received 194,650 citations, corresponding to an unadjusted citations-per-paper value of 14.47.

**Figure 3:**
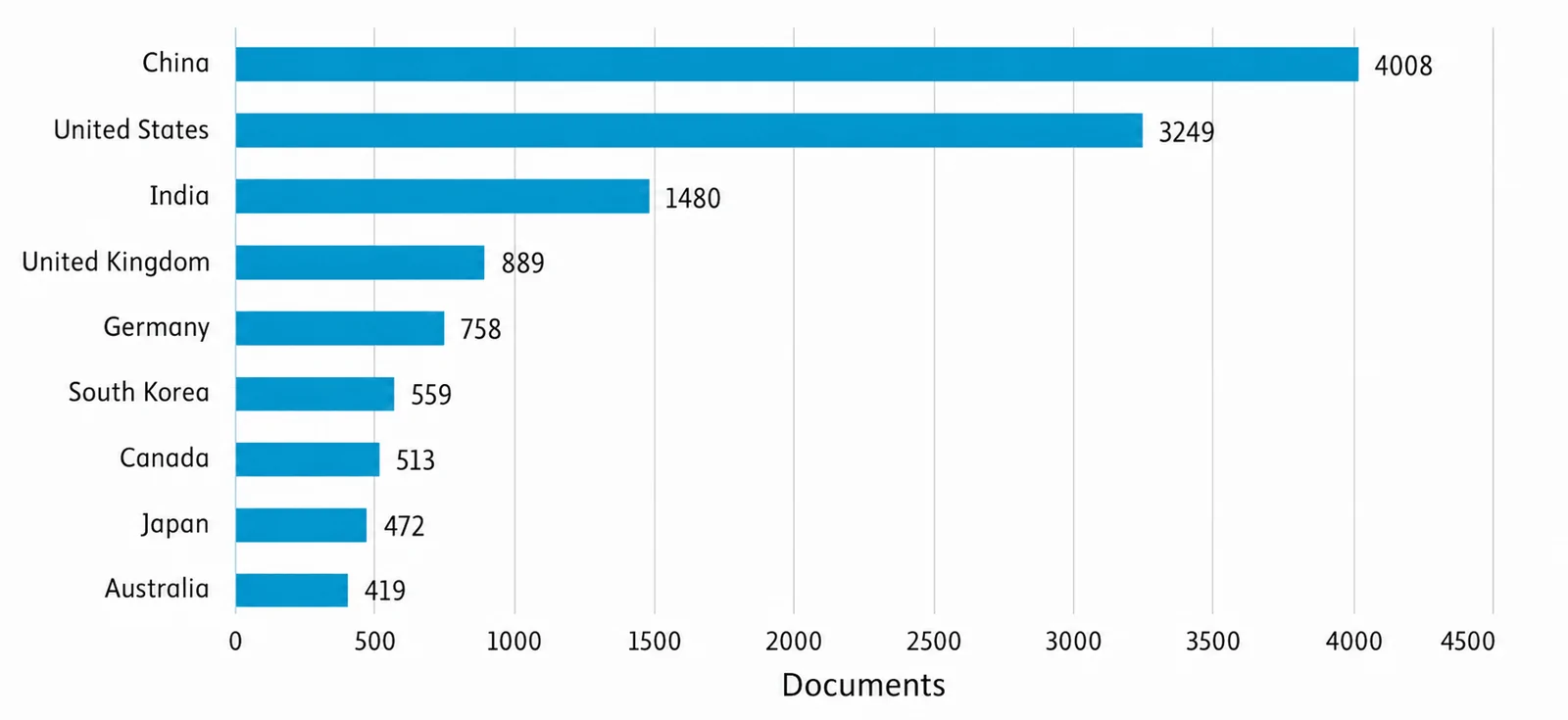
Country-wise distribution of global research output in generative and foundation-based artificial intelligence applied to medical imaging Bars represent the number of publications associated with each country under full counting. Multinational publications were attributed to every country represented in the author affiliations; country counts are therefore non-mutually exclusive. The chart was generated using Microsoft Excel for Microsoft 365, 2026 (Microsoft Corporation, Redmond, WA, USA), from bibliographic data exported from Scopus.

Country-level publication and citation indicators are presented in Figure 3 and Table 1. China had the largest publication output, followed by the United States, India, and the United Kingdom. The United States received the highest total number of citations, whereas Canada had the highest unadjusted citations per paper among the nine countries presented. The United Kingdom ranked fourth by publication volume and had an unadjusted citations-per-paper value of 21.00. Country counts were derived using full counting and were therefore non-mutually exclusive.

**Table 1:** Contribution of the top nine countries to global research output in medical imaging, with comparison of publication volume and citation impact metrics relative to the United Kingdom Metrics include total publications (TP), total citations (TC), citations per paper (CPP), and percentage representation within the global dataset (TP%). TP% was calculated using the global total number of records (n=13,452) as the denominator. Under full counting, each multinational publication was attributed to every country represented in its author affiliations. TP and TP% values are therefore non-mutually exclusive and may sum to more than the number of unique publications or 100%. Values are descriptive bibliometric indicators; no inferential statistical testing was performed.

Rank	Name of country	TP	TC	CPP	TP (%)
1	China	4008	52,130	13.00	29.8
2	United States	3249	73,310	22.56	24.2
3	India	1480	11,155	7.54	11.0
4	United Kingdom	889	18,672	21.00	6.6
5	Germany	758	11,208	14.78	5.6
6	South Korea	559	10,612	18.98	4.2
7	Canada	513	14,404	28.07	3.8
8	Japan	472	5999	12.71	3.5
9	Australia	419	10,576	25.25	3.1

United Kingdom Publication and Citation Trends

Annual UK-affiliated output increased from 17 publications in 2017 to 305 in 2025, representing an approximately 18-fold increase (Table 2). From 2019 onwards, the UK generally contributed approximately 6%-7% of annual global output. More than one-third of all UK-affiliated publications in the dataset were published in 2025.

**Table 2:** Annual trends in global and United Kingdom-affiliated research output in generative and foundation-based artificial intelligence in medical imaging (2017-2025) Global TP represents the annual number of Scopus-indexed records in the global dataset. UK TP represents the annual number of records with at least one United Kingdom-affiliated author. TP of global (%) was calculated using annual global TP as the denominator for each year. TP of UK (%) was calculated using the total UK-affiliated dataset as the denominator (n=889). UK TC represents total citations received by UK-affiliated publications in each year. CPP was calculated as TC divided by UK TP. YoY growth was calculated as (current-year UK TP - previous-year UK TP/previous-year UK TP) x 100. The 2017 YoY value was not applicable because it was the first year in the study period. Values are descriptive bibliometric indicators; no inferential statistical testing was performed. CPP, citations per paper; TC, total citations; TP, total publications; YoY, year-on-year.

Period	Global TP	UK TP	TP of global (%)	TP of UK (%)	UK TC	UK CPP	YoY growth (%)
2017	131	17	13.0	1.9	1098	64.59	-
2018	256	20	7.8	2.3	1947	97.35	17.7
2019	525	35	6.7	3.9	1503	42.94	75.0
2020	753	48	6.4	5.4	2472	51.50	37.1
2021	1011	69	6.8	7.8	2970	43.04	43.8
2022	1138	75	6.6	8.4	1716	22.88	8.7
2023	1690	120	7.1	13.5	3463	28.86	60.0
2024	3038	200	6.6	22.5	2638	13.19	66.7
2025	4910	305	6.2	34.3	865	2.84	52.5
Total	13,452	889	-	-	18,672	-	-

Unadjusted citations per paper were higher for earlier publication years and lower for recent years, consistent with the shorter citation window available to newer publications. Annual growth in UK publication output was variable but accelerated again from 2023 onwards.

Document Types

**Figure 4:**
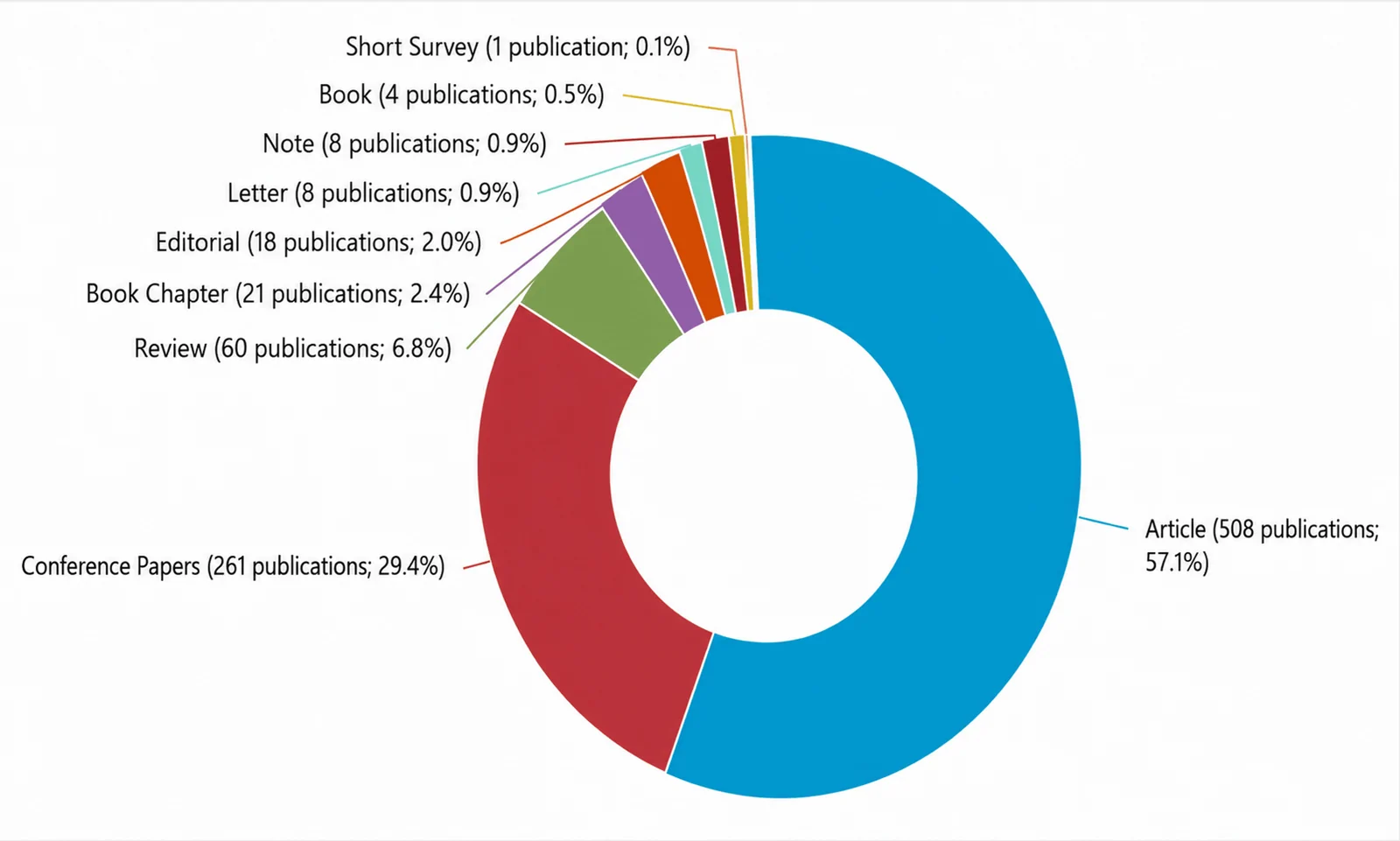
Distribution of document types among United Kingdom-affiliated publications in generative and foundation-based artificial intelligence applied to medical imaging Proportions are shown for journal articles, conference papers, reviews, and other document types indexed in Scopus, with percentages calculated using the total UK-affiliated publication denominator (n=889). The chart was generated using Microsoft Excel for Microsoft 365, 2026 (Microsoft Corporation, Redmond, WA, USA), from bibliographic data exported from Scopus.

Journal articles constituted the majority of UK-affiliated publications, followed by conference papers and reviews (Figure 4). Comparative document-type distributions are presented in Supplementary Material 1. The United States had the highest proportion of journal articles among the four leading countries, while India had the highest proportion of conference papers. The UK profile included a majority of journal articles, alongside a substantial conference-paper component.

Institutional Productivity

The eight most productive UK organisations are presented in Table 3. Imperial College London had the largest publication output, followed by King’s College London, University College London, and the University of Oxford. The National Heart and Lung Institute had the highest unadjusted citations per paper among the organisations presented. Institution-level counts were based on full counting and were therefore non-mutually exclusive.

**Table 3:** The eight most productive United Kingdom organisations in generative and foundation-based artificial intelligence in medical imaging research, ranked by total publications TP% represents the proportion of UK-affiliated publications contributed by each institution, calculated using the total UK-affiliated dataset as the denominator (n=889). Values are descriptive bibliometric indicators; no inferential statistical testing was performed. Eight organisations are presented because incomplete or unresolvable affiliation metadata prevented reliable identification of the top 10 organisations. CPP, citations per paper; TC, total citations; TP, total publications.

Rank	Name of organisation	TP	TP (%)	TC	CPP
1	Imperial College London	131	14.7	3310	25.27
2	King’s College London	110	12.4	3462	31.47
3	University College London	101	11.4	4171	41.30
4	University of Oxford	97	10.9	3666	37.79
5	University of Cambridge	75	8.4	1689	22.52
6	The University of Edinburgh	38	4.3	1254	33.00
7	National Heart and Lung Institute	37	4.2	1713	46.30
8	University of Leeds	33	3.7	721	21.85

Author Productivity and Collaboration

A total of 3942 authors contributed to the UK-affiliated dataset. Author-level productivity, citation impact, and collaboration metrics are presented in Tables 4, 5. Yang G had both the highest publication count and the highest total link strength among the listed authors, while Alexander DC had the highest unadjusted citations per paper.

**Table 4:** The 10 most productive authors in the United Kingdom-affiliated generative and foundation-based artificial intelligence in medical imaging research and their collaboration strength TP% represents the proportion of UK-affiliated publications associated with each author, calculated using the total UK-affiliated dataset as the denominator (n=889). TLS was calculated in VOSviewer and represents the overall strength of co-authorship links for each author within the analysed network. Because publications may include multiple authors, author publication counts are not mutually exclusive, and percentages may sum to more than 100%. Values are descriptive bibliometric indicators; no inferential statistical testing was performed. CPP, citations per paper; TC, total citations; TLS, total link strength; TP, total publications.

Rank	Name of author	Name of organisation	TP	TP (%)	TC	CPP	TLS
1	Yang G	Imperial College London	42	4.7	1965	46.79	154
2	Schnabel JA	King’s College London	16	1.8	81	5.06	57
3	Frangi AF	University of Manchester	14	1.6	96	6.86	52
4	Rueckert D	Imperial College London	14	1.6	90	6.43	24
5	Ourselin S	King’s College London	13	1.5	847	65.15	41
6	Tsaftaris SA	University of Edinburgh	13	1.5	496	38.15	28
7	Alexander DC	University College London	11	1.2	1114	101.27	15
8	Botchu R	The Royal Orthopaedic Hospital NHS Foundation Trust	11	1.2	208	18.91	25
9	Huang J	National Heart and Lung Institute	11	1.2	280	25.45	72
10	Iyengar KP	Southport and Ormskirk Hospital NHS Trust	11	1.2	208	18.91	25

**Table 5:** Top 10 most connected authors in the co-authorship network based on collaboration strength in generative and foundation-based artificial intelligence in medical imaging research Authors are ranked by TLS, derived from VOSviewer co-authorship network analysis. TP represents the absolute number of publications associated with each author in the Scopus-exported UK-affiliated dataset. TLS represents the overall strength of co-authorship links for each author within the analysed network. Because publications may include multiple authors, author-level publication counts are not mutually exclusive. Values are descriptive bibliometric indicators; no inferential statistical testing was performed. TLS, total link strength; TP, total publications.

Rank	Author	TP	TLS
1	Yang G	42	154
2	Wang C	7	79
3	Huang J	11	72
4	Zhang Y	8	62
5	Schnabel JA	16	57
6	Pinaya WHL	9	54
7	Frangi AF	14	52
8	Zhang J	13	51
9	Hu Y	8	48
10	Cardoso MJ	8	45

**Figure 5:**
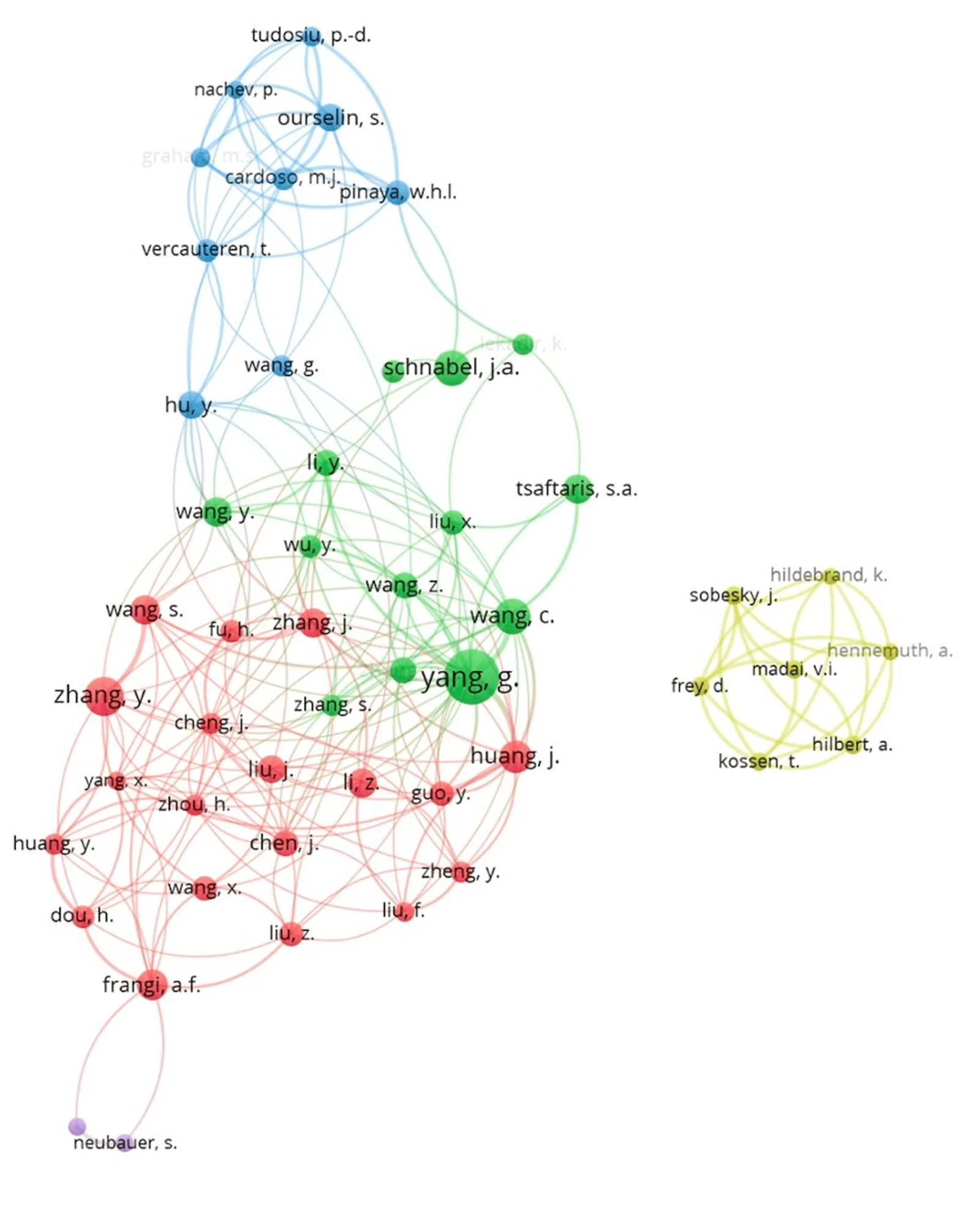
Co-authorship network of authors publishing on generative and foundation-based artificial intelligence in medical imaging, with emphasis on generative and foundation-based methods Node size represents publication volume, link thickness indicates co-authorship strength, and colours denote collaboration clusters identified through VOSviewer network analysis. Authors positioned closer together have stronger co-authorship relationships within the analysed dataset. The network shows a large interconnected component on the left, containing several overlapping collaboration clusters, and a smaller separate component on the right, indicating a more distinct co-authorship group with limited linkage to the main network. Only authors meeting the predefined inclusion threshold were visualised. The network was generated using full counting in VOSviewer version 1.6.19, released in 2023.

For the co-authorship analysis, 327 authors met the minimum threshold of three publications (Supplementary Material 2), and the 50 authors with the highest total link strength were visualised (Figure 5). The network comprised five clusters, including one smaller component that was relatively separate from the principal interconnected network.

External Funding

Of the 889 UK-affiliated publications, 552 (62.1%) reported at least one external funding source. A total of 160 funder-name entries were identified as indexed in Scopus. Because funder names were not normalised and umbrella organisations were retained alongside constituent councils, these entries should not be interpreted as 160 unique funding organisations.

**Figure 6:**
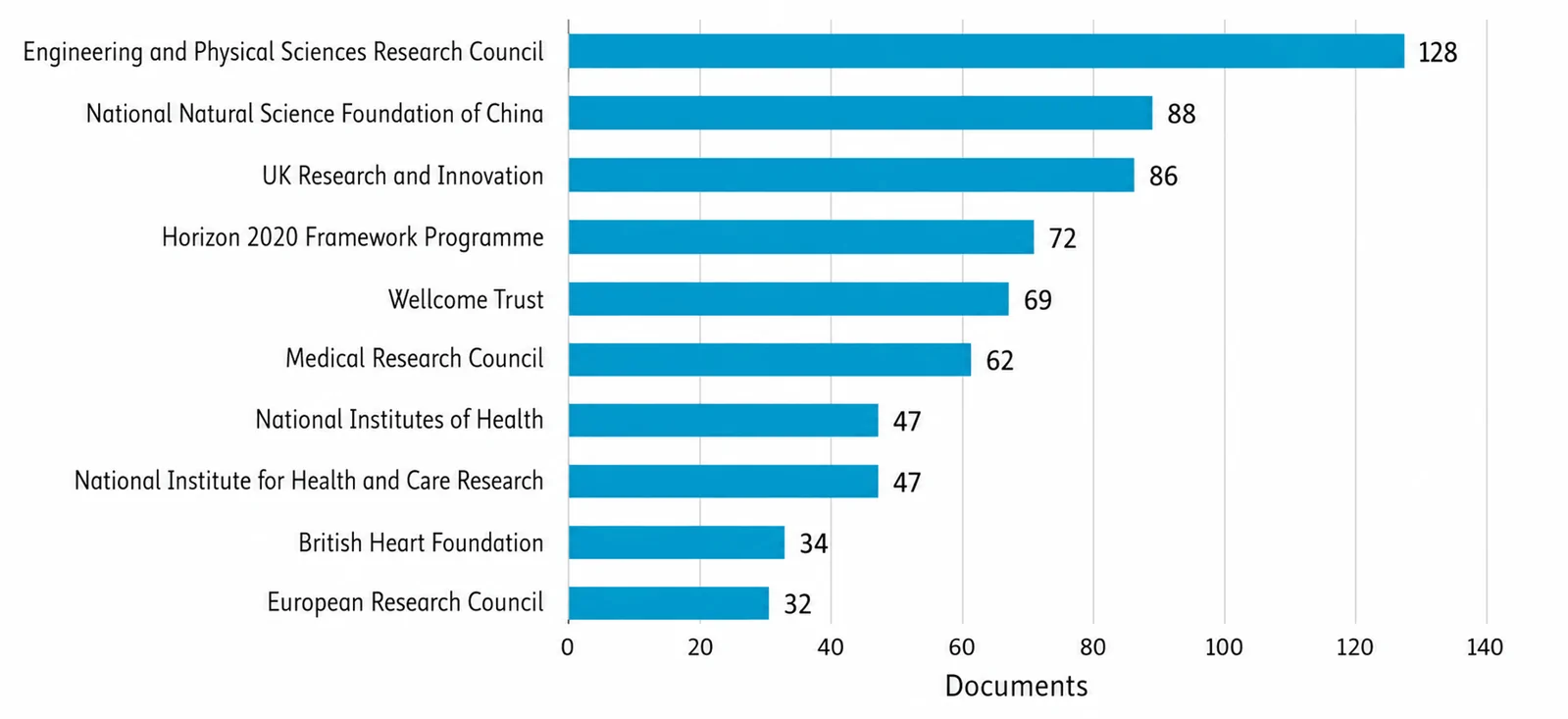
Leading funding organisations acknowledged in United Kingdom-affiliated publications on generative and foundation-based artificial intelligence in medical imaging Bars represent the number of Scopus-indexed documents reporting financial support from each funding body. The chart was generated using Microsoft Excel for Microsoft 365, 2026 (Microsoft Corporation, Redmond, WA, USA), from bibliographic data exported from Scopus.

The Engineering and Physical Sciences Research Council was the most frequently acknowledged funder, followed by the National Natural Science Foundation of China, UK Research and Innovation, the Horizon 2020 Framework Programme, the Wellcome Trust, and the Medical Research Council (Figure 6). Publications could acknowledge more than one funder.

Leading Publishing Sources

The UK-affiliated publications were distributed across 160 indexed sources. The 20 most productive sources accounted for 337 publications, or 37.9% of the UK dataset. Lecture Notes in Computer Science had the largest publication output, while NeuroImage and IEEE Transactions on Medical Imaging had among the highest unadjusted citations-per-paper values.

Keyword Analysis

A total of 7685 keywords were identified. After application of the minimum occurrence threshold, 332 keywords met the threshold (Supplementary Material 3). After exclusion of selected generic indexing terms (Supplementary Material 4) and ranking by frequency, 40 keywords were included in the network analysis (Supplementary Material 5). The most frequent terms were “deep learning”, “medical imaging”, “diagnostic imaging”, and “GANs.”

**Figure 7:**
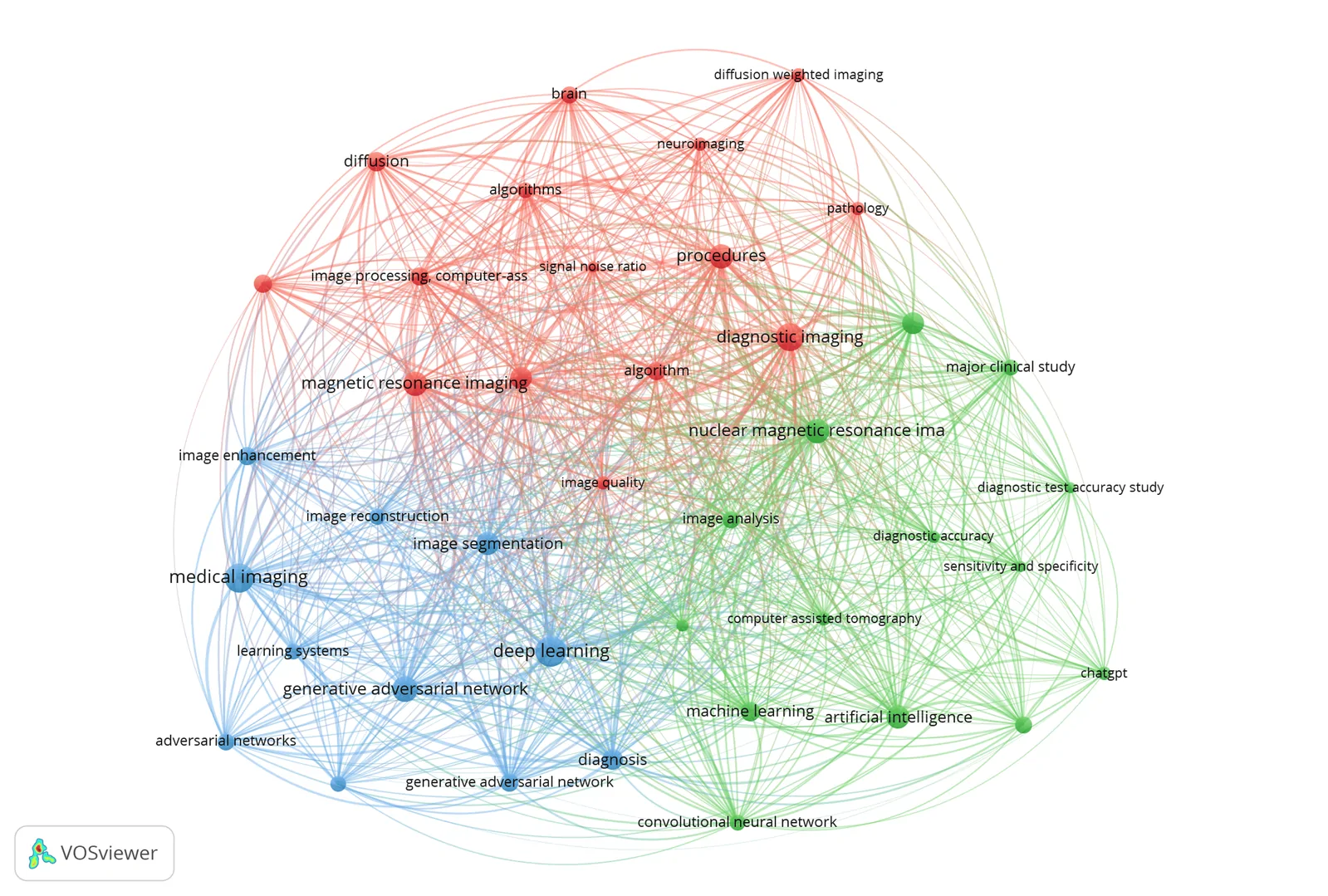
Keyword co-occurrence network of United Kingdom-affiliated publications in generative and foundation-based artificial intelligence applied to medical imaging Node size reflects keyword frequency, link thickness indicates co-occurrence strength, and node colour denotes thematic clusters. Keywords positioned closer together co-occurred more frequently within the same publications. The blue cluster primarily represents generative and deep learning methods applied to medical imaging, including generative adversarial networks, deep learning, image reconstruction, image enhancement, and image segmentation. The red cluster is centred on magnetic resonance imaging and diffusion-related applications, including diffusion, diffusion-weighted imaging, neuroimaging, image quality, algorithms, and diagnostic imaging. The green cluster represents broader artificial intelligence and diagnostic evaluation themes, including artificial intelligence, machine learning, convolutional neural networks, image analysis, diagnostic accuracy, sensitivity and specificity, major clinical studies, and ChatGPT.

The co-occurrence network comprised three principal thematic clusters (Figure 7). The blue cluster was centred on generative and deep-learning methods, including GANs, medical imaging, reconstruction, enhancement, and segmentation. The red cluster primarily represented MRI, diffusion-related imaging, neuroimaging, image quality, and diagnostic imaging. The green cluster included broader AI, machine learning, radiology, image analysis, ChatGPT, large language models, and diagnostic-evaluation terms.

Highly Cited Publications

The 20 most highly cited UK-affiliated publications are presented in Table 6. Together, they received 5389 citations. The most highly cited publication was the 2018 DAGAN study by Yang et al, followed by studies of MedGAN, retinal foundation models, NiftyNet, and GANs for medical image analysis.

**Table 6:** The 20 most highly cited United Kingdom-affiliated publications in generative and foundation-based artificial intelligence applied to medical imaging, with emphasis on generative and foundation-based methods Publications are ranked by total citation count in the Scopus-exported dataset. Citation counts represent citations accrued through 31 December 2025, as recorded in Scopus. The approximate annual citation rate was calculated by dividing total citations by the number of calendar years from the publication year through 2025 inclusive (2025−publication year+1). This year-based calculation did not account for the exact publication month or date and should therefore be interpreted as an approximate rather than exact annualised rate. Mean annual citations during the first five calendar years were calculated by summing citations accrued in the publication year and the subsequent four calendar years and dividing by five. N/A indicates that a complete five-calendar-year citation window was unavailable by the end of 2025. Reference numbers in the title column correspond to the full reference list. Values are descriptive bibliometric indicators; no inferential statistical testing was performed.

Rank	Citations	Average citations per year	Average citations per year in first five years	Author citation	Year	Title
1	923	115.38	138.6	Yang et al [15]	2018	DAGAN: Deep De-Aliasing Generative Adversarial Networks for Fast Compressed Sensing MRI Reconstruction
2	508	84.67	97	Armanious et al [16]	2020	MedGAN: medical image translation using GANs
3	492	164	N/A	Zhou et al [17]	2023	A foundation model for generalizable disease detection from retinal images
4	478	59.75	58	Gibson et al [18]	2018	NiftyNet: a deep-learning platform for medical imaging
5	364	60.67	71.4	Kazeminia et al [19]	2020	GANs for medical image analysis
6	297	148.5	N/A	Huang et al [20]	2024	Segment anything model for medical images?
7	271	67.75	N/A	Wyatt et al [21]	2022	AnoDDPM: Anomaly Detection with Denoising Diffusion Probabilistic Models using Simplex Noise
8	218	54.5	N/A	Pinaya et al [22]	2022	Brain imaging generation with latent diffusion models
9	202	28.86	36.2	Han et al [23]	2019	Combining noise-to-image and image-to-image GANs: brain MR image augmentation for tumour detection
10	193	64.33	N/A	Jiang et al [24]	2023	Deep Learning for Medical Image-Based Cancer Diagnosis
11	183	26.14	30.4	Ben-Cohen et al [25]	2019	Cross-modality synthesis from CT to PET using FCN and GAN networks for improved automated lesion detection
12	170	34	34	Han et al [26]	2021	MADGAN: unsupervised medical anomaly detection GAN using multiple adjacent brain MRI slice reconstruction
13	165	18.33	22	Chartsias et al [27]	2017	Adversarial image synthesis for unpaired multi-modal cardiac data
14	163	32.6	32.6	Ma et al [28]	2021	Structure and illumination constrained GAN for medical image enhancement
15	144	24	28.8	Dar et al [29]	2020	Prior-guided image reconstruction for accelerated multi-contrast MRI via generative adversarial networks
16	138	69	N/A	Wu et al [30]	2024	MedSegDiff-V2: diffusion-based medical image segmentation with transformer
17	125	17.86	22.4	Rau et al [31]	2019	Implicit domain adaptation with conditional generative adversarial networks for depth prediction in endoscopy
18	121	24.2	24.2	Karakanis et al [32]	2020	Lightweight deep learning models for detecting COVID-19 from chest X-ray images
19	118	59	N/A	Dayarathna et al [33]	2024	Deep learning-based synthesis of MRI, CT and PET: Review and analysis
20	116	16.57	19	Han et al [34]	2019	Synthesizing diverse lung nodules wherever massively: 3D multi-conditional GAN-based CT image augmentation for object detection

Earlier highly cited publications were predominantly concerned with GAN-based reconstruction, synthesis, augmentation, and image analysis, whereas the more recent highly cited set included diffusion and foundation-model architectures. Because citation rankings are strongly influenced by publication age, this descriptive pattern should not be interpreted as definitive evidence of a thematic transition.

Discussion

Principal Findings

This bibliometric analysis characterised global and United Kingdom-affiliated research on generative and foundation-based AI in medical imaging between 2017 and 2025. The findings position the UK as a major contributor to the field, with comparatively high unadjusted citation impact, strong participation from research-intensive institutions, extensive co-authorship, and a thematic profile spanning generative methods, diffusion-related imaging, and broader diagnostic applications.

The United Kingdom ranked fourth by full-count publication volume and had an unadjusted citations-per-paper value of 21.00. This suggests that UK-affiliated work achieved substantial visibility relative to several higher-output countries. However, such comparisons should be interpreted cautiously because raw citation indicators are affected by publication age, document type, disciplinary composition, collaboration patterns, and highly cited outliers.

The institutional and author-level findings indicate that UK activity is anchored in a group of research-intensive organisations and interconnected collaborative networks. This is consistent with the multidisciplinary nature of medical imaging AI, which commonly requires expertise in clinical imaging, computer science, engineering, statistics, and data governance. Differences in institutional citation impact further show that productivity and influence are related but distinct dimensions of research performance.

The mixture of journal articles and conference papers reflects the dual clinical and technical character of the field. Journal publication remains important for clinical and translational dissemination, while conference proceedings continue to provide a major route for rapidly evolving methodological research. Cross-country differences in publication format may therefore reflect different disciplinary and dissemination structures rather than differences in research quality alone.

The funding profile similarly highlights the interdisciplinary basis of UK-affiliated research. Support from engineering and computational funders alongside biomedical, charitable, European, and international organisations suggests that progress in this area depends on combined investment in methodological development, clinical evaluation, and translational infrastructure. The presence of both domestic and international funders also reflects the collaborative and cross-border nature of medical imaging AI research.

Comparison With Previous Studies

The sustained increase in publication volume mirrors broader bibliometric analyses showing rapid expansion of AI research in medicine and radiology since 2019 [10]. Generative and deep learning methods were prominent within this growth, reflected by frequent keywords such as “deep learning”, “medical imaging”, “diagnostic imaging”, and “GANs”, as well as by GAN-, diffusion-, and foundation model-related papers among highly cited UK-affiliated publications. This is consistent with a previous top-cited analysis in which deep learning was the most frequent keyword and neuroimaging and oncology were prominent clinical subjects [35]. A recent India-focused bibliometric analysis of generative AI in healthcare imaging similarly identified rapid national research growth, supporting the relevance of country-level bibliometric mapping [14].

The keyword and highly cited publication analyses also suggest thematic evolution over time. Earlier highly cited publications were predominantly concerned with GAN-based reconstruction, synthesis, augmentation, anomaly detection, and cross-modality translation, particularly in MRI [15,16,19,23,25-29]. More recent highly cited publications included foundation models, general-purpose segmentation approaches, diffusion-based models, and multimodal synthesis reviews [17,20-22,30,33]. However, because citation rankings are strongly influenced by publication age, this pattern should not be interpreted as definitive evidence of a temporal transition in the wider literature.

Clinical and Translational Implications

The prominence of MRI-related applications may reflect the suitability of MRI for reconstruction, denoising, synthesis, acceleration, and image-quality enhancement. These applications are clinically relevant because acquisition time, image quality, and workflow efficiency remain important practical challenges. However, bibliometric visibility reflects research attention rather than evidence of methodological quality, routine clinical adoption, or patient benefit. These outcomes require direct evaluation through clinical and implementation studies.

Deep learning reconstruction has shown potential to reduce MRI acquisition times [36], while workforce shortages and rising imaging demand provide a relevant service context [37]. However, prospective evaluation and deployment guidance remain necessary before routine clinical implementation [38,39].

Implementation may be affected by data governance, interoperability, computational infrastructure, procurement, regulatory evaluation, model monitoring, and accountability [40-42]. Generative imaging models introduce concerns relating to artefact generation, image authenticity, and deepfakes [43,44]. Medically intended AI software may also fall within medical device regulation [45]. Standardised evaluation and transparent reporting are therefore important, with CONSORT-AI and TRIPOD+AI providing relevant guidance for applicable study designs [46,47].

The concentration of UK research within major academic centres and its dense collaboration networks may support multicentre validation and coordinated evaluation. The identified areas of research activity, including MRI reconstruction, image enhancement, segmentation, multimodal imaging, and foundation models, provide potential priorities for further study, although their clinical value must be assessed against patient need, safety, workflow integration, cost-effectiveness, and equity.

UK Policy and Funding Implications

The concentration of output within research-intensive institutions indicates an established UK academic base but also suggests potential value in broadening participation across NHS and university centres. Future funding could support multicentre datasets, cross-institutional validation, and collaboration among NHS imaging departments, universities, industry, regulators, and patient representatives. This is consistent with the AI Opportunities Action Plan, which emphasises infrastructure, data access, public sector adoption, and translation of AI capability into societal benefit [48].

The interdisciplinary funding profile suggests that UK medical imaging AI already spans engineering, computational, and biomedical research. UK Research and Innovation funding programmes for AI in biomedical and health research provide a relevant model through their emphasis on partnerships, translational impact, and reusable outputs [49]. Medical Research Council priorities concerning AI and related technologies further reflect the strategic importance of AI within biomedical research [50].

Secure imaging datasets, trusted research environments, computational capacity, interoperability, and clear governance pathways are likely to influence translation. NHS information-governance guidance emphasises lawful data use, transparency, accountability, and appropriate governance [40], while Medicines and Healthcare products Regulatory Agency (MHRA) guidance indicates that medically intended software and AI may fall within medical device regulation [45]. Funding and policy support should therefore balance development of novel models with validation, governance, monitoring, and evaluation across NHS settings.

Limitations

This study has several limitations inherent to bibliometric analysis. The analysis was restricted to publications indexed in Scopus and may not have captured relevant records indexed exclusively in other databases. Scopus was selected because of its broad coverage of engineering, computer science, biomedical, medical imaging, and conference proceedings literature, which represent central dissemination routes for medical imaging AI research. Although adding databases such as Web of Science or PubMed could increase record capture, cross-database merging introduces challenges, including metadata inconsistencies, duplicate records, and discordant citation counts. A single-database approach was therefore adopted to preserve internal consistency and reproducibility. Although 2025 represented a completed calendar year, indexing of publications released late in 2025 may still have been incomplete when the Scopus data were retrieved in February 2026. Consequently, publication and citation counts for 2025 may be underestimated.

The findings were also dependent on the chosen search strategy. Although the strategy was designed to capture generative and foundation-based AI methods in medical imaging, terminology in this field is heterogeneous and rapidly evolving. The search was focused rather than exhaustive and relied on broad umbrella concepts and dominant model families. Consequently, some publications indexed exclusively under narrower terms, such as “variational autoencoder”, “VAE”, “vision-language model”, or “multimodal foundation model”, may not have been retrieved.

Conversely, broad terms such as “GAN”, “ChatGPT”, and “Gemini AI” may have retrieved records outside the intended scope. Although manual review of a stratified random sample of 400 records indicated a strict estimated precision of 90.0%, not every retrieved record underwent individual relevance screening, and some false-positive or indeterminate records may therefore remain.

Citation-based indicators were unadjusted for publication age, document type, research field, collaboration patterns, and highly cited outliers. Citations per paper may therefore be skewed and should be interpreted as a descriptive indicator rather than a normalised measure of research quality or comparative impact. Citations per year provided partial adjustment for publication age among highly cited publications but did not account for field or document-type differences. Citation counts may also be affected by journal visibility, article type, self-citation, field size, and collaboration structure.

The study relied on bibliographic metadata and did not assess the technical performance, clinical implementation, regulatory status, ethical characteristics, or patient-level impact of individual AI models. Full rather than fractional counting may overrepresent contributions from multi-authored or multi-institutional publications. Affiliation-based filtering may also underrepresent UK researchers contributing to international collaborations where their UK affiliations were not indexed.

Finally, keyword co-occurrence and co-authorship maps are sensitive to threshold selection, author name disambiguation, indexing quality, and VOSviewer settings. The absence of systematic synonym merging may have divided occurrences across equivalent terms, influenced keyword frequencies, and cluster composition.

## Conclusions

This bibliometric analysis demonstrates rapid global growth in generative and foundation-based AI research applied to medical imaging between 2017 and 2025. UK-affiliated work represented a substantial component of the field and had a comparatively high unadjusted mean citation rate. The UK ranked fourth by publication volume and showed relatively high citation visibility, with research activity concentrated among major academic centres and supported by interdisciplinary funding. Keyword and highly cited publication analyses indicate prominent themes around generative and deep learning methods, MRI reconstruction and enhancement, diffusion-based approaches, and foundation-model architectures. These findings provide a transparent bibliometric reference point for monitoring the evolution of AI medical imaging research while recognising that citation-based indicators do not directly measure clinical implementation, methodological quality, or patient-level impact.

## Appendices

Supplementary material 1

VOSviewer Parameters

For both networks in Figures 5, 7, the clustering resolution was set to 1.00, the minimum cluster size to one item, and a fixed seed of 1 was specified for both the advanced layout and advanced clustering parameters. The attraction parameter was set to 2 for both networks, while the repulsion parameter was set to −1 for the co-authorship network (Figure 5) and 0 for the keyword co-occurrence network (Figure 7). All remaining advanced clustering and layout parameters were retained at their default settings.

**Table 7:** Document-type distribution of publications from the four leading countries by research output Values are presented as the number of publications and the percentage of each country’s total output. Journal articles and conference papers are shown separately, while “other document types” includes all remaining Scopus-indexed categories, such as reviews, book chapters, editorials, letters, notes, books, and short surveys. Percentages were calculated using the total number of publications for each country as the denominator and may not sum to exactly 100% because of rounding. Country-level publication counts were derived using full counting; multinational publications were therefore credited to each country represented in the author affiliations. n, number of publications.

Country	Total publications, n	Journal articles, n (%)	Conference papers, n (%)	Other document types, n (%)
China	4008	2172 (54.2%)	1279 (31.9%)	557 (13.9%)
United States	3249	2118 (65.2%)	932 (28.7%)	199 (6.1%)
India	1480	568 (38.4%)	620 (41.9%)	292 (19.7%)
United Kingdom	889	508 (57.1%)	261 (29.4%)	120 (13.5%)

 Supplementary material 2 

**Table 8:** Total link strength of all authors meeting the minimum publication threshold in the UK-affiliated co-authorship network. Authors are ranked in descending order of total link strength (TLS). TLS represents the cumulative strength of an author’s co-authorship links with other authors in the VOSviewer network. Higher TLS values indicate greater overall collaborative connectivity within the analysed dataset. The table includes all 327 authors who met the predefined threshold of at least three publications. Authors with a TLS of 0 met the publication threshold but had no co-authorship links with other eligible authors in the final network. Author names are presented as indexed in the Scopus-exported dataset. TLS, total link strength.

Rank	Author	TLS	Rank	Author	TLS	Rank	Author	TLS
1	yang, g.	154	110	li, l.	19	219	wagner, f.	10
2	wang, c.	79	111	reynaud, h.	19	220	wang, m.	10
3	huang, j.	72	112	yang, l.	19	221	wei, h.	10
4	zhang, y.	62	113	zhang, l.	19	222	wise, r.g.	10
5	schnabel, j.a.	57	114	zhang, q.	19	223	wood, d.	10
6	pinaya, w.h.l.	54	115	aviles-rivero, a.i.	18	224	ying, m.t.c.	10
7	frangi, a.f.	52	116	blackledge, m.d.	18	225	baugh, m.	9
8	zhang, j.	51	117	burrage, m.k.	18	226	butala, m.d.	9
9	hu, y.	48	118	chen, c.	18	227	clarkson, m.j.	9
10	cardoso, m.j.	45	119	ferreira, v.m.	18	228	guo, x.	9
11	wang, s.	44	120	gao, z.	18	229	lio', p.	9
12	liu, j.	41	121	huang, z.	18	230	lu, y.	9
13	ourselin, s.	41	122	küstner, t.	18	231	puyol-antón, e.	9
14	wang, x.	40	123	messiou, c.	18	232	qin, c.	9
15	chen, j.	38	124	popescu, i.a.	18	233	sanchez, p.	9
16	frey, d.	37	125	sharma, h.	18	234	shang, k.	9
17	hilbert, a.	37	126	zhao, y.	18	235	shen, y.	9
18	kossen, t.	37	127	zheng, s.	18	236	slabaugh, g.	9
19	li, y.	37	128	zhu, j.	18	237	tian, h.	9
20	madai, v.i.	37	129	alvarez-valle, j.	17	238	zhang, k.	9
21	sobesky, j.	37	130	chen, h.	17	239	zhang, z.	9
22	cheng, j.	35	131	dombrowski, m.	17	240	zhao, h.	9
23	huang, y.	35	132	gibson, e.	17	241	zhao, m.	9
24	li, z.	35	133	jenko, n.	17	242	zhou, j.	9
25	wu, y.	35	134	li, w.	17	243	zhou, m.	9
26	dou, h.	34	135	murao, k.	17	244	chandrashekar, a.	8
27	lekadir, k.	34	136	pérez-garcía, f.	17	245	han, l.	8
28	liu, x.	34	137	richardson, h.	17	246	handa, a.	8
29	hennemuth, a.	33	138	wetscherek, m.t.a.	17	247	jiang, l.	8
30	hildebrand, k.	33	139	firmin, d.	16	248	kather, j.n.	8
31	yang, x.	33	140	gómez, a.	16	249	kerfoot, e.	8
32	zheng, y.	33	141	li, q.	16	250	li, h.	8
33	zhou, h.	32	142	macgillivray, t.j.	16	251	liu, q.	8
34	wang, z.	31	143	macnaught, g.	16	252	liu, w.	8
35	fu, h.	30	144	modat, m.	16	253	lukasiewicz, t.	8
36	graham, m.s.	30	145	newby, d.e.	16	254	papageorghiou, a.t.	8
37	liu, f.	30	146	rees, g.	16	255	pournik, o.	8
38	liu, z.	30	147	sala, e.	16	256	xu, y.	8
39	zhang, h.	30	148	sun, y.	16	257	yan, y.	8
40	nachev, p.	29	149	winfield, j.m.	16	258	zhou, t.	8
41	vercauteren, t.	29	150	wu, f.	16	259	zhou, t.	8
42	guo, y.	28	151	xia, y.	16	260	abbasi, s.f.	6
43	neubauer, s.	28	152	diaz, o.	26	261	afsar minhas, f.	6
44	piechnik, s.k.	28	153	kainz, b.	26	262	alexander, d.c.	15
45	tsaftaris, s.a.	28	154	ni, d.	26	263	ali, h.	5
46	tudosiu, p.-d.	28	155	schönlieb', c.-b.	26	264	ali, s.	4
47	wang, g.	28	156	yang, b.	26	265	arvanitis, t.n.	11
48	wang, y.	28	157	barratt, d.c.	26	266	bernabéu, m.o.	0
49	noble, j.a.	27	158	botchu, r.	25	267	booth, t.c.	11
50	osuala, r.	27	159	iyengar, k.p.	25	268	botnar, r.	11
51	ravikumar, n.	27	160	lee, a.g.	25	269	chamabara, n.	10
52	yang, y.	27	161	masalkhi, m.	25	270	chen, k.	6
53	zhang, s.	27	162	ong, j.	25	271	chen, l.	5
54	aviles-rivero, a.i.	18	163	tavakoli, a.	25	272	chen, m.	2
55	chen, c.	18	164	waisberg, e.	25	273	christiaens, d.	0
56	huang, z.	18	165	botchu, r.	25	274	clark, c.a.	0
57	messiou, c.	18	166	fabijan, a.	24	275	denker, a.	2
58	popescu, i.a.	18	167	fabijan, r.	24	276	deshpande, s.	6
59	zheng, s.	18	168	han, c.	24	277	dey, s.	3
60	zhu, j.	18	169	li, c.	24	278	figini, m.	6
61	chen, j.	38	170	nowosławska, e.	24	279	galinović, i.	27
62	cheng, j.	35	171	polis, b.	24	280	glocker, b.	4
63	dou, h.	34	172	rueckert, d.	24	281	hammers, a.	3
64	hennemuth, a.	33	173	wang, h.	24	282	harmon, s.a.	4
65	hildebrand, k.	33	174	wu, x.	24	283	he, j.	7
66	fu, h.	30	175	zhang, x.	24	284	he, y.	6
67	liu, f.	30	176	ravikumar, n.	27	285	hu, x.	13
68	liu, z.	30	177	fiebach, j.b.	27	286	ianuş, a.	4
69	zhang, h.	30	178	galinović, i.	27	287	jimmy, j.	20
70	nachev, p.	29	179	khalil, a.a.	27	288	jones, d.k.	0
71	vercauteren, t.	29	180	noble, j.a.	27	289	kalantar, r.	14
72	guo, y.	28	181	osuala, r.	27	290	kamnitsas, k.	10
73	neubauer, s.	28	182	yang, y.	27	291	king, a.p.	7
74	piechnik, s.k.	28	183	zhang, s.	27	292	kunze, k.p.	11
75	tsaftaris, s.a.	28	184	nachev, p.	29	293	lam, k.	4
76	tudosiu, p.-d.	28	185	vercauteren, t.	29	294	lamata, p.	4
77	wang, g.	28	186	diaz, o.	26	295	leemans, a.	0
78	wang, y.	28	187	kainz, b.	26	296	li, g.	11
79	noble, j.a.	27	188	ni, d.	26	297	li, r.	14
80	osuala, r.	27	189	schönlieb', c.-b.	26	298	li, s.	10
81	ravikumar, n.	27	190	yang, b.	26	299	lin, c.	3
82	yang, y.	27	191	botchu, r.	25	300	liu, c.	12
83	zhang, s.	27	192	iyengar, k.p.	25	301	liu, h.	11
84	diaz, o.	26	193	lee, a.g.	25	302	lu, x.	2
85	kainz, b.	26	194	masalkhi, m.	25	303	ma, x.	7
86	ni, d.	26	195	ong, j.	25	304	maier-hein, l.	3
87	schönlieb', c.-b.	26	196	tavakoli, a.	25	305	marias, k.	7
88	yang, b.	26	197	waisberg, e.	25	306	mills, r.	13
89	botchu, r.	25	198	fabijan, a.	24	307	mishra, d.	13
90	iyengar, k.p.	25	199	fabijan, r.	24	308	montana, g.	7
91	lee, a.g.	25	200	han, c.	24	309	murray-smith, r.	0
92	masalkhi, m.	25	201	li, c.	24	310	nischal, n.	11
93	ong, j.	25	202	nowosławska, e.	24	311	papanikolaou, n.	7
94	tavakoli, a.	25	203	polis, b.	24	312	patel, a.	12
95	waisberg, e.	25	204	rueckert, d.	24	313	patitucci, e.	10
96	fabijan, a.	24	205	wang, h.	24	314	perelli, a.	7
97	fabijan, r.	24	206	wu, x.	24	315	petersen, s.e.	15
98	han, c.	24	207	zhang, x.	24	316	puglisi, l.	5
99	li, c.	24	208	ariaryatne, s.	20	317	qiu, j.	5
100	nowosławska, e.	24	209	chen, z.	20	318	rajpoot, n.	6
101	polis, b.	24	210	gao, y.	20	319	ravì, d.	5
102	rueckert, d.	24	211	jimmy, j.	20	320	rekik, i.	0
103	wang, h.	24	212	lv, j.	20	321	rundo, l.	28
104	wu, x.	24	213	nan, y.	20	322	saeed, s.u.	13
105	zhang, x.	24	214	punwani, s.	20	323	satoh, s.	14
106	ariaryatne, s.	20	215	wu, w.	20	324	stoyanov, d.	0
107	chen, z.	20	216	xing, x.	20	325	trucco, e.	1
108	gao, y.	20	217	zakrzewski, k.	20	326	wardlaw, j.	1
109	jimmy, j.	20	218	yang, l.	19	327	zia, t.	3

Supplementary material 3 

**Table 9:** Keywords meeting the minimum occurrence threshold for the VOSviewer co-occurrence analysis. The table presents all Scopus-indexed keywords occurring at least 10 times in the UK-affiliated dataset, together with their number of occurrences and total link strength. Occurrences indicate the number of publications in which each keyword appeared, while total link strength represents the cumulative strength of that keyword’s co-occurrence links with other keywords in the network. Generic indexing terms were retained in this complete supplementary list but were excluded from the final visualised network where specified in Table 10.

Keyword	Occurrences	Total link strength
3d reconstruction	10	86
accuracy	21	411
adolescent	14	302
adult	102	2075
adversarial learning	11	165
adversarial machine learning	20	237
adversarial networks	102	1129
aged	63	1322
aged, 80 and over	10	225
algorithm	116	2263
algorithms	87	1743
alzheimer disease	21	460
alzheimer's disease	10	205
alzheimer's disease	10	180
anatomical structures	12	142
animal	14	272
animals	14	272
anisotropy	12	225
anomaly detection	16	210
apparent diffusion coefficient	17	394
area under the curve	15	314
article	326	6177
artifact	19	377
artificial intelligence	172	2513
artificial neural network	49	968
attention	15	253
auto encoders	18	255
autoencoder	23	519
automation	12	217
benchmarking	38	594
bioinformatics	11	155
biological marker	11	211
biomedical imaging	13	190
brain	94	1874
brain mapping	25	465
brain MRI	15	222
brain neoplasms	10	251
brain region	12	264
brain tumor	25	513
brain tumors	11	133
breast cancer	18	305
breast neoplasms	10	197
breast tumor	11	223
cancer diagnosis	15	313
cardiac imaging	12	230
cardiac mri	15	218
cardiovascular magnetic resonance	18	378
chatgpt	65	894
child	13	226
classification	12	236
classification (of information)	32	457
classifier	11	237
clinical article	32	600
clinical decision making	23	376
clinical evaluation	13	266
clinical practice	15	268
clinical research	20	259
cohort analysis	42	851
comparative study	42	878
computational linguistics	13	122
computed tomography	21	278
computer aided diagnosis	23	351
computer assisted diagnosis	36	830
computer assisted tomography	65	1227
computer model	21	365
computer simulation	18	375
computer vision	42	555
computerized tomography	88	1011
condition	10	79
connectome	12	260
contrast medium	13	256
contrastive learning	20	287
controlled study	154	3150
convolution	15	235
convolutional neural network	85	1456
convolutional neural networks	49	621
coronavirus disease 2019	13	200
covid-19	15	219
cross validation	10	222
cross-sectional study	12	263
data accuracy	10	156
data augmentation	42	521
data privacy	15	206
data processing	10	217
databases, factual	13	308
de-noising	30	431
decision making	36	545
deep learning	291	4253
deep neural network	21	473
deep neural networks	25	392
denoising diffusion probabilistic model	11	156
diagnosis	146	2032
diagnostic accuracy	55	1058
diagnostic imaging	243	4831
diagnostic radiography	12	89
diagnostic test accuracy study	42	874
diagnostic value	10	180
diffusion	119	1795
diffusion magnetic resonance imaging	39	829
diffusion model	106	1221
diffusion models	27	350
diffusion mri	16	281
diffusion tensor imaging	23	409
diffusion weighted imaging	61	1282
disease classification	12	264
disease progression	13	202
diseases	40	551
domain adaptation	10	133
down-stream	10	190
drift diffusion model	12	176
dynamic contrast enhanced mri	12	147
echocardiography	14	232
echography	17	377
editorial	19	230
electronic health record	14	166
endoscopy	17	253
factual database	13	308
feasibility study	13	285
feature extraction	33	647
federated learning	14	178
female	135	2836
fluid-attenuated inversion recovery imaging	13	335
follow up	15	262
forecasting	20	282
foundation model	13	240
foundation models	42	521
fractional anisotropy	11	228
functional magnetic resonance imaging	26	430
gadolinium	12	234
gallium nitride	11	41
gan	31	332
gans	11	100
gaussian noise (electronic)	11	174
generative adversarial network	113	1751
generative adversarial network (gan)	10	131
generative adversarial networks	204	2507
generative artificial intelligence	35	514
generative model	56	765
generative models	17	250
glioma	11	241
gray matter	17	394
health care	23	255
health care personnel	12	151
healthcare	10	144
heart	24	379
high resolution	13	115
histology	17	335
histopathology	29	518
human	437	7719
human experiment	24	504
human tissue	24	553
humans	303	5789
image analysis	102	2045
image classification	20	264
image coding	18	211
image denoising	22	353
image enhancement	104	1496
image fusion	10	101
image generations	20	261
image interpretation, computer-assisted	29	714
image processing	149	3047
image processing, computer-assisted	111	2398
image quality	60	1309
image reconstruction	77	1175
image registration	14	218
image resolution	11	152
image segmentation	171	2721
image synthesis	25	297
image translation	16	206
images reconstruction	18	260
images segmentations	21	289
images synthesis	34	415
imaging	13	222
imaging phantom	10	187
imaging, three-dimensional	22	495
information processing	20	384
intermethod comparison	12	298
inverse problems	21	204
iterative methods	14	158
language	16	208
language model	57	519
large dataset	23	353
large datasets	16	230
large language model	95	1323
large language models	32	410
learn+	12	132
learning	30	595
learning algorithm	20	432
learning algorithms	14	202
learning models	17	210
learning systems	80	1042
lung	11	209
lung cancer	20	262
machine learning	125	2098
machine-learning	31	349
magnetic resonance	32	448
magnetic resonance imaging	191	3184
major clinical study	85	1766
male	136	2836
mathematical model	10	197
mean square error	12	123
measurement accuracy	10	256
medical applications	12	106
medical computing	33	303
medical education	19	199
medical image analysis	32	449
medical image processing	43	491
medical image segmentation	28	374
medical imaging	264	2857
metabolism	12	190
metadata	10	163
microstructure	10	193
middle aged	48	1031
model	10	213
motion	13	274
mri	31	480
mri reconstruction	14	163
multi-modal	32	384
multimodal imaging	16	350
natural language processing	31	515
natural language processing systems	14	128
neoplasm	11	224
nerve cell network	21	422
network architecture	14	231
neural networks	22	294
neural networks, computer	42	839
neural-networks	16	221
neurodegenerative diseases	25	406
neuroimaging	63	1207
noise	13	287
nonhuman	17	327
normal human	16	368
nuclear magnetic resonance	15	195
nuclear magnetic resonance imaging	187	3712
ophthalmology	23	313
optical coherence tomography	18	293
optical resolving power	20	231
pathology	62	1172
pathophysiology	10	196
patient care	15	214
patient treatment	14	134
performance	49	701
personalized medicine	13	236
physiology	22	424
population statistics	10	139
positron emission tomography	27	539
practice guideline	10	129
prediction	40	814
predictive value	22	434
priority journal	28	584
probabilistic models	19	219
procedures	193	3997
prognosis	10	219
prospective studies	10	199
prospective study	14	277
prostate cancer	17	315
qualitative analysis	14	292
quality control	28	487
quantitative analysis	33	676
radiography	10	186
radiologist	17	299
radiology	46	586
radiology reports	13	107
radiotherapy	10	164
reaction time	11	167
receiver operating characteristic	25	532
reliability	12	227
report generation	12	114
reproducibility	22	482
reproducibility of results	17	356
retina	13	235
retrospective studies	11	244
retrospective study	30	660
review	48	760
risk assessment	11	188
scoring system	10	195
segmentation	19	334
self-supervised learning	19	317
semantic segmentation	19	218
semantics	23	322
semi-supervised learning	10	175
sensitivity analysis	11	197
sensitivity and specificity	43	921
signal noise ratio	38	873
signal to noise ratio	18	231
signal-to-noise ratio	14	334
simulation	28	493
state of the art	12	120
superresolution	12	134
supervised learning	13	178
supervised machine learning	15	361
support vector machine	14	254
surgery	13	209
synthesis	15	233
synthetic data	30	406
synthetic images	15	182
systematic review	14	247
t1 weighted imaging	21	547
t2 weighted imaging	18	456
textures	14	162
therapy	10	171
thorax radiography	19	336
three dimensional computer graphics	14	212
three-dimensional imaging	31	733
tissue	16	203
tomography, optical coherence	10	170
tomography, x-ray computed	24	460
training	31	572
training data	10	101
transfer learning	18	248
transfer of learning	12	286
transformer	10	156
treatment planning	15	221
tumors	27	377
ultrasonic imaging	13	91
ultrasonics	14	173
ultrasonography	11	236
ultrasound	18	314
ultrasound images	13	168
uncertainty	10	103
united kingdom	12	198
unsupervised anomaly detection	11	110
urology	12	157
validation process	10	190
very elderly	10	225
vision	13	269
visual languages	10	93
white matter	23	488
workflow	11	176
x ray	14	264
x-ray computed tomography	36	619
young adult	18	417

Supplementary material 4 

**Table 10:** Generic indexing terms excluded from the keyword co-occurrence analysis Occurrences represent the number of records in which each indexed keyword appeared. These generic demographic and publication-type terms were excluded before keyword co-occurrence network analysis because they did not provide meaningful thematic information.

Keyword excluded from keyword analysis	Occurrences
Human	437
Article	326
Humans	303
Male	136
Female	135
Adult	102
Aged	63
Middle aged	48

Supplementary material 5 

**Table 11:** Keyword co-occurrence clusters identified in the VOSviewer analysis of United Kingdom-affiliated publications on generative and foundation-based artificial intelligence in medical imaging. Keywords are grouped according to the three clusters generated algorithmically by VOSviewer. Occurrences represent the number of publications in which each keyword appeared within the analysed dataset. The thematic cluster labels were assigned by the authors through qualitative interpretation of the dominant keywords within each cluster and were not generated automatically by VOSviewer. Full counting was used.

Cluster	Suggested thematic label	Keyword	Occurrences
1	MRI, diffusion imaging and image-processing applications	Diagnostic imaging	243
1	MRI, diffusion imaging and image-processing applications	Procedures	193
1	MRI, diffusion imaging and image-processing applications	Magnetic resonance imaging	191
1	MRI, diffusion imaging and image-processing applications	Image processing	149
1	MRI, diffusion imaging and image-processing applications	Algorithm	116
1	MRI, diffusion imaging and image-processing applications	Diffusion	119
1	MRI, diffusion imaging and image-processing applications	Image processing, computer-assisted	111
1	MRI, diffusion imaging and image-processing applications	Diffusion model	106
1	MRI, diffusion imaging and image-processing applications	Image quality	60
1	MRI, diffusion imaging and image-processing applications	Brain	94
1	MRI, diffusion imaging and image-processing applications	Algorithms	87
1	MRI, diffusion imaging and image-processing applications	Neuroimaging	63
1	MRI, diffusion imaging and image-processing applications	Pathology	62
1	MRI, diffusion imaging and image-processing applications	Diffusion weighted imaging	61
1	MRI, diffusion imaging and image-processing applications	Signal noise ratio	38
2	Clinical evaluation, artificial intelligence and diagnostic performance	Nuclear magnetic resonance imaging	187
2	Clinical evaluation, artificial intelligence and diagnostic performance	Artificial intelligence	172
2	Clinical evaluation, artificial intelligence and diagnostic performance	Controlled study	154
2	Clinical evaluation, artificial intelligence and diagnostic performance	Machine learning	125
2	Clinical evaluation, artificial intelligence and diagnostic performance	Image analysis	102
2	Clinical evaluation, artificial intelligence and diagnostic performance	Large language model	95
2	Clinical evaluation, artificial intelligence and diagnostic performance	Computerized tomography	88
2	Clinical evaluation, artificial intelligence and diagnostic performance	Major clinical study	85
2	Clinical evaluation, artificial intelligence and diagnostic performance	Convolutional neural network	85
2	Clinical evaluation, artificial intelligence and diagnostic performance	Computer assisted tomography	65
2	Clinical evaluation, artificial intelligence and diagnostic performance	ChatGPT	65
2	Clinical evaluation, artificial intelligence and diagnostic performance	Diagnostic accuracy	55
2	Clinical evaluation, artificial intelligence and diagnostic performance	Artificial neural network	49
2	Clinical evaluation, artificial intelligence and diagnostic performance	Sensitivity and specificity	43
2	Clinical evaluation, artificial intelligence and diagnostic performance	Diagnostic test accuracy study	42
3	Deep learning, generative adversarial networks and image reconstruction	Deep learning	291
3	Deep learning, generative adversarial networks and image reconstruction	Medical imaging	264
3	Deep learning, generative adversarial networks and image reconstruction	Generative adversarial networks	204
3	Deep learning, generative adversarial networks and image reconstruction	Image segmentation	171
3	Deep learning, generative adversarial networks and image reconstruction	Generative adversarial network	113
3	Deep learning, generative adversarial networks and image reconstruction	Image enhancement	104
3	Deep learning, generative adversarial networks and image reconstruction	Diagnosis	146
3	Deep learning, generative adversarial networks and image reconstruction	Image reconstruction	77
3	Deep learning, generative adversarial networks and image reconstruction	Learning systems	80
3	Deep learning, generative adversarial networks and image reconstruction	Adversarial networks	102

## References

[1] Yu KH, Beam AL, Kohane IS (2018). Artificial intelligence in healthcare. Nat Biomed Eng.

[2] Wang S, Zhou X, Li C, Wang S, Li Y, Tan T, Zheng H (2025). Generative artificial intelligence in medical imaging: foundations, progress, and clinical translation. Research.

[3] Sordo Z, Chagnon E, Hu Z (2025). Synthetic scientific image generation with VAE, GAN, and diffusion model architectures. J Imaging.

[4] Kim S, Park H, Park SH (2024). A review of deep learning-based reconstruction methods for accelerated MRI using spatiotemporal and multi-contrast redundancies. Biomed Eng Lett.

[5] Fabian Z, Heckel R, Soltanolkotabi M (2021). Data augmentation for deep learning based accelerated MRI reconstruction with limited data. Proc Int Conf Mach Learn.

[6] Gupta P, Ding B, Guan C, Ding D (2024). Generative AI: a systematic review using topic modelling techniques. Data and Information Management.

[7] Senthil R, Anand T, Somala CS, Saravanan KM (2024). Bibliometric analysis of artificial intelligence in healthcare research: trends and future directions. Future Healthc J.

[8] Öztürk O, Kocaman R, Kanbach DK (2024). How to design bibliometric research: an overview and a framework proposal. Rev Manag Sci.

[9] Chogtu B, V R, Naik A, Dutta S, Venkata SK (2025). Exploring the bibliometric impact of artificial intelligence in radiology: an analytical approach. Int J Inf Manag Data Insights.

[10] Lin M, Lin L, Lin L, Lin Z, Yan X (2025). A bibliometric analysis of the advance of artificial intelligence in medicine. Front Med.

[11] Baalla A, Jellouli T (2026). Bibliometric analysis of artificial intelligence in medical imaging for hospital performance and regulation. Discov Artif Intell.

[12] (2026). GOV.UK: Fit for the future: 10 year health plan for England (accessible version). https://www.gov.uk/government/publications/10-year-health-plan-for-england-fit-for-the-future/fit-for-the-future-10-year-health-plan-for-england-accessible-version.

[13] Hong SJ, Yoon DY, Lim KJ, Moon JY, Yoon SJ, Seo YL, Yun EJ (2026). Department of Health & Social Care: A guide to good practice for digital and data-driven health technologies. Journal of the Belgian Society of Radiology.

[14] Vaishya R, Gupta BM, M CS, Guechairi S, Vaish A, Botchu R (2025). Indian research on generative artificial intelligence in healthcare imaging: a comprehensive bibliometric analysis. J Clin Imaging Sci.

[15] Yang G, Yu S, Dong H (2018). DAGAN: deep de-aliasing generative adversarial networks for fast compressed sensing MRI reconstruction. IEEE Trans Med Imaging.

[16] Armanious K, Jiang C, Fischer M (2020). MedGAN: medical image translation using GANs. Comput Med Imaging Graph.

[17] Zhou Y, Chia MA, Wagner SK (2023). A foundation model for generalizable disease detection from retinal images. Nature.

[18] Gibson E, Li W, Sudre C (2018). NiftyNet: a deep-learning platform for medical imaging. Comput Methods Programs Biomed.

[19] Kazeminia S, Baur C, Kuijper A, van Ginneken B, Navab N, Albarqouni S, Mukhopadhyay A (2020). GANs for medical image analysis. Artif Intell Med.

[20] Huang Y, Yang X, Liu L (2024). Segment anything model for medical images?. Med Image Anal.

[21] Wyatt J, Leach A, Schmon SM, Willcocks CG (2022). AnoDDPM: anomaly detection with denoising diffusion probabilistic models using simplex noise. IEEE Xplore.

[22] Pinaya WHL, Tudosiu PD, Dafflon J (2022). Brain imaging generation with latent diffusion models. Deep Generative Models.

[23] Han H, Rundo L, Araki R (2019). Combining noise-to-image and image-to-image GANs: brain MR image augmentation for tumor detection. IEEE Access.

[24] Jiang X, Hu Z, Wang S, Zhang Y (2023). Deep learning for medical image-based cancer diagnosis. Cancers.

[25] Ben-Cohen A, Klang E, Raskin SP (2019). Cross-modality synthesis from CT to PET using FCN and GAN networks for improved automated lesion detection. Eng Appl Artif Intell.

[26] Han C, Rundo L, Murao K (2021). MADGAN: unsupervised medical anomaly detection GAN using multiple adjacent brain MRI slice reconstruction. BMC Bioinformatics.

[27] Chartsias A, Joyce T, Dharmakumar R, Tsaftaris SA (2017). Adversarial image synthesis for unpaired multi-modal cardiac data. Simulation and Synthesis in Medical Imaging.

[28] Ma Y, Liu J, Liu Y (2021). Structure and illumination constrained GAN for medical image enhancement. IEEE Trans Med Imaging.

[29] Dar SUH, Yurt M, Shahdloo M, Ildiz ME, Tinaz B, Cukur T (2020). Prior-guided image reconstruction for accelerated multi-contrast MRI via generative adversarial networks. EEE J Sel Top Signal Process.

[30] Wu J, Ji W, Fu H, Xu M, Jin Y, Xu Y (2024). MedSegDiff-V2: diffusion-based medical image segmentation with transformer. Proc AAAI Conf Artif Intell.

[31] Rau A, Edwards PJ, Ahmad OF, Riordan P, Janatka M, Lovat LB, Stoyanov D (2019). Implicit domain adaptation with conditional generative adversarial networks for depth prediction in endoscopy. Int J Comput Assist Radiol Surg.

[32] Karakanis S, Leontidis G (2021). Lightweight deep learning models for detecting COVID-19 from chest X-ray images. Comput Biol Med.

[33] Dayarathna S, Islam KT, Uribe S, Yang G, Hayat M, Chen Z (2024). Deep learning based synthesis of MRI, CT and PET: review and analysis. Medical Image Analysis.

[34] Han C, Kitamura Y, Kudo A (2019). Synthesizing diverse lung nodules wherever massively: 3D multi-conditional GAN-based CT image augmentation for object detection. IEEE Xplore.

[35] Hughes H, O'Reilly M, McVeigh N, Ryan R (2023). The top 100 most cited articles on artificial intelligence in radiology: a bibliometric analysis. Clin Radiol.

[36] Rastogi A, Brugnara G, Foltyn-Dumitru M (2024). Deep-learning-based reconstruction of undersampled MRI to reduce scan times: a multicentre, retrospective, cohort study. Lancet Oncol.

[37] (2026). The Royal College of Radiologists: Clinical radiology workforce census 2024. https://www.rcr.ac.uk/media/4imb5jge/_rcr-2024-clinical-radiology-workforce-census-report.pdf.

[38] (2026). The Royal College of Radiologists: AI deployment fundamentals for medical imaging. https://www.rcr.ac.uk/our-services/all-our-publications/clinical-radiology-publications/ai-deployment-fundamentals-for-medical-imaging/.

[39] (2026). NHS England: Artificial intelligence (AI) and machine learning. https://www.england.nhs.uk/long-read/artificial-intelligence-ai-and-machine-learning/.

[40] (2026). NHS England: Artificial intelligence guidance for IG professionals. https://digital.nhs.uk/data-and-information/information-governance/guidance/artificial-intelligence/guidance-for-ig-professionals.

[41] (2026). NHS England: Digital Technology Assessment Criteria (DTAC) guidance for buyers and suppliers. https://digital.nhs.uk/services/digital-technology-assessment-criteria-dtac.

[42] (2026). NHS England: Interoperability. https://www.england.nhs.uk/long-read/interoperability/.

[43] Xia M, Bayerlein R, Chemli Y (2026). On hallucinations in artificial intelligence-generated content for nuclear medicine imaging (the DREAM report). J Nucl Med.

[44] Pradeepan P, Raj S G, George J (2025). A comprehensive review of deepfakes in medical imaging: ethical concerns, detection techniques and future directions. Appl Comput Sci.

[45] (2026). GOV.UK: Software and artificial intelligence (AI) as a medical device. https://www.gov.uk/government/publications/software-and-artificial-intelligence-ai-as-a-medical-device/software-and-artificial-intelligence-ai-as-a-medical-device.

[46] Liu X, Cruz Rivera S, Moher D, Calvert MJ, Denniston AK (2020). Reporting guidelines for clinical trial reports for interventions involving artificial intelligence: the CONSORT-AI extension. Nat Med.

[47] Collins GS, Moons KG, Dhiman P (2024). TRIPOD+AI statement: updated guidance for reporting clinical prediction models that use regression or machine learning methods. BMJ.

[48] (2026). GOV.UK: AI opportunities action plan. https://www.gov.uk/government/publications/ai-opportunities-action-plan/ai-opportunities-action-plan.

[49] (2026). UK Research and Innovation: Artificial intelligence for better biomedical and health research. https://www.ukri.org/opportunity/artificial-intelligence-for-better-biomedical-and-health-research/.

[50] (2026). UK Research and Innovation: Artificial intelligence, engineering biology and quantum technologies: highlight notice. https://www.ukri.org/opportunity/artificial-intelligence-engineering-biology-and-quantum-technologies-highlight-notice/.

